# Comparing the Primary and Recall Immune Response Induced by a New EV71 Vaccine Using Systems Biology Approaches

**DOI:** 10.1371/journal.pone.0140515

**Published:** 2015-10-14

**Authors:** Jie Shao, Junnan Zhang, Xing Wu, Qunying Mao, Pan Chen, Fengcai Zhu, Miao Xu, Wei Kong, Zhenglun Liang, Junzhi Wang

**Affiliations:** 1 School of Life Sciences, Jilin University, Changchun, Jilin, P.R.China; 2 Division of Hepatitis Virus Vaccines, National Institutes for Food and Drug Control, Beijing, P.R.China; 3 Jiangsu Provincial Center for Disease Control and Prevention, Nanjing, Jiangsu, P.R.China; University of Massachusetts Medical School, UNITED STATES

## Abstract

Three inactivated EV71 whole-virus vaccines have completed Phase III clinical trials in mainland China, with high efficacy, satisfactory safety, and sustained immunogenicity. However, the molecular mechanisms how this new vaccine elicit potent immune response remain poorly understood. To characterize the primary and recall responses to EV71 vaccines, PBMC from 19 recipients before and after vaccination with EV71 vaccine are collected and their gene expression signatures after stimulation with EV71 antigen were compared. The results showed that primary and recall response to EV71 antigen have both activated an IRF7 regulating type I interferon and antiviral immune response network. However, up-regulated genes involved in T cell activation regulated by IRF1, inflammatory response, B-cell activation and humoral immune response were only observed in recall response. The specific secretion of IL-10 in primary response and IL-2,IP-10,CCL14a, CCL21 in recall response was consistent with the activation of immune response process found in genes. Furthermore, the expression of MX1 and secretion of IP-10 in recall response were strongly correlated with NTAb level at 180d after vaccination (r = 0.81 and 0.99). In summary, inflammatory response, adaptive immune response and a stronger antiviral response were indentified in recall response.

## Introduction

Hand foot and mouth disease (HFMD) is a serious public health problem in Western Pacific region countries[1]. From May 2008 to March 2015, 11.96 million cases of HFMD had been reported in mainland China, of which 3,227 were fatal[2]. Based on the epidemiological and clinical etiological data published in recent years, more than 80% of the pathogens isolated from patients died from HFMD were identified as enterovirus 71 (EV71)[3–6]. There are no efficient drugs available for EV71 treatment, sovaccines will be the essential way to control the EV71 outbreak. Research and development of EV71 vaccine was carried out in several Asian countries. Currently three inactivated EV71 whole-virus vaccines in mainland China have completed Phase III clinical trials in more than 30,000 infants and children. Results showed that these vaccines were safe and there were over 90% efficacy in preventing EV71-associated HFMD, 80% efficacy in preventing EV71-associated diseases[7–9]. However, a comprehensive understanding of immune responses to this new vaccine is still lacking.

Recently, systems biology approach has been used to predict the development of protective immunity after vaccination by profiling gene expression of PBMC samples from vaccinated individuals. This approach has been pioneered in the studies of yellow fever vaccine[10–11],influenza vaccine[12] and HPV virus-like particles vaccine[13]. In these studies, genes involved in innate immune response pathways were enriched after vaccination, and specific biomarkers were found to predict the immune effect of vaccines with high accuracy[10–13]. Since most of these researches were focused on the primary responses after initial immunization, little is known about the recall response. The recall response is important due to its close relationship with neutralizing antibodies (NTAb) titer and immune persistence[14].

In this study, microarray analysis and cytokine profiling have been performed to compare gene expression patterns between primary and recall immune response induced by EV71 vaccines. Our results provide a better understanding of the immune response induced by EV71 vaccine.

## Materials and Methods

### 1. Study design

Participants were selected from a randomized, double-blind, placebo-controlled phase III trial of inactivated EV71 whole-virus vaccines, which was conducted in a sample of 10245 healthy children aged 6–35 months (ClinicalTrials.gov, number NCT01508247)[7]. This clinical trial study was approved by the institutional review board of Jiangsu Provincial Center of Disease Control and Prevention, and all guardians of participants provided written informed consent. The immunization schedule consisted of two doses given on day 0 and day 28. Vaccine or placebo was administered intramuscularly to the anterolateral side of the thigh (for participants aged 6–11 months) or the deltoid muscle (those aged 12–35 months). Blood specimens were collected before the initial dose (day 0) and day 56 and 180 after the first vaccination. Plasma and peripheral blood mononuclear cells (PBMCs) were separated on the scene and frozen at -80°C.

Nineteen vaccine recipients whose antibody level is negative before vaccination and seroconversion on day 56 after vaccination were selected in this study. However, 5 samples pre-vaccination and 3 samples post-vaccination were excluded from the microarray detection because of poor RNA purity and integrity.Only day 0 and 56 samples were selected for this study because earlier findings indicated that the largest increases in cytokine responses were typically observed at month 2[15]. Thirteen vaccine recipients (5 on day 0 and 8 on day 56) were selected for protein array analysis.

### 2. Inactivated EV71 whole-virus vaccine and EV71 liquid bulk

Inactivated alum-adjuvanted EV71 whole-virus vaccine (vero cell), containing 320U of antigen and 0.18mg of alum, was developed by Beijing Vigoo Biological with a seed virus of EV71 strain FY7VP5/AH/CHN/2008 (subgenotype C4). Each dose of placebo contains 0.18mg of alum adjuvant and no EV71 antigen.

EV71 liquid bulk for stimulation in vitro contained 4000U/ml of antigen and no alum.

### 3. PBMCs incubation and RNA extraction

Cryopreserved PBMCs were thawed and cultured. PBMCs (10*10^6^) were plated in each well of a 6-well plate (Costar) in RPMI 1640 medium supplemented with penicillin-streptomycin (100g/ml-100U/ml; Invitrogen), L-glutamine (2mM), HEPES buffer (10mM), and 10% heat-inactivated FCS (HyClone). Cells were cultured for 24h at 37°C with EV71 Ag (200u/ml). Media was used as a background measurement for untreated cells. A total of four incubations were set up on the same day for each subject. The order of sample preparation was randomly defined from the list of selected subjects. When harvesting, cultures were centrifuged and cell precipitation were frozen at -80°C.

Total RNA extracts were performed using an RNeasy Total RNA isolation kit (Qiagen). RLT lysis buffer was quickly added to the culture well and then to the cell pellet to include both adherent and suspension cells. RNA purity and integrity were tested by microcapillary electrophoresis using the Agilent 2100 bioanalyzer (Agilent Technologies). The sample of RIN>6 was selected for follow-up experiment.

### 4. Microarray analysis

Microarray gene expression analysis was performed using a human genome 70-mer oligonucleotide microarray (CapitalBio Corporation 35K Human Genome Array),that contains 35035 probesets to 25100 characterized human genes. Total RNA preparation and labeled cDNA synthesis and hybridization were performed according to the manufacturer’s recommended protocol (CapitalBio). In short, 1 ug total RNA was used for double-stranded cDNA (ds-cDNA) synthesis with the CbcScript reverse transcriptase kit (Capitalbio) with the T7 Oligo (dT). Klenow enzyme labeling strategy was adopted after reverse transcription using CbcScript Ⅱ reverse transcriptase. Labeled cDNA were hybridized performed in a CapitalBio BioMixer^TM^ II Hybridization Station and washed in a Capitalbio SlideWasher^TM^ (CapitalBio) with two consecutive solutions. Arrays were scanned with a confocal LuxScan^TM^ scanner and the images obtained were then analyzed using LuxScan^TM^ 3.0 software (CapitalBio). After background subtracted in both channels (Cy3 and Cy5),faint spots were extracted for which intensities were exceed 400 units. A space- and intensity-dependent normalization based on a LOWESS program was employed[16]. To determine the significant differentially expressed genes, Significance Analysis of Microarrays (SAM, version 3.02) were performed.

### 5. Quantitative real-time PCR and confirmation of microarray results

The gene expression levels were measured by RT-PCR. A total of 17 genes in 60 samples were selected for confirmation. The primers are listed in Table A in S1 File. Briefly, total RNA was extracted from the PBMCs as described above. For quantification, a single-tube RT-PCR assay was performed using the TaqMan 1-step RT-PCR Master Mix in a 7500 Fast Real-time RT-PCR system. The following protocol was used for all PCR assays: 5 min at 42°C and 10s at 95°C,followed by 40 cycles at 95°C for 5s and 60°C for 30s.

### 6. Protein array analysis

Cryopreserved PBMCs were thawed and cultured as the previously described in ‘Microarray analysis’ of Materials and Methods. PBMCs were cultured in serum-free medium (Dakewei) for 48h at 37°C with EV71 Ag (200u/ml) and medium was used for control. Cell culture supernatants were harvested and protein concentration were tested according to the manufacturer’s recommended protocol of BCATM Protein Array Kit (Pierce). Each sample was concentrated four times and then detected by protein array, according to the protocol of RayBio^®^Human Cytokine Antibody Array G Series 4000.

### 7. Neutralizing Antibody

EV71-neutralizing Ab titers were determined using classical cytopathic effect (CPE) assay performed as previously described[17]. Plasma samples were inactivated at 56°C for 30 minutes,serially diluted two fold from 1:8 and mixed with equal volumes of TCID50 of a EV71 strain. The mixture was dispensed into a 96-well microplate and incubated at 37°C for 2 hours. RD cells (1–2*10^5^cells/mL) were added to the mixture. The plates were then placed in a CO_2_ incubator at 35°C for 7 days. CPE was observed by microscopy. EV71 national standards were included in each test as a control for the reproducibility of the results[18]. Neutralizing antibody titers of EV71 were defined as the dilution rate showing 50% inhibition of the CPE. Undetectable Ab levels were considered as 4. All individuals selected for the microarray analysis had no detectable Abs before vaccination. After vaccination, all individuals developed Ab titers against EV71 in the group of vaccination.

## Results

### 1. The neutralizing antibodies response to EV71 vaccine

**Fig 1:**
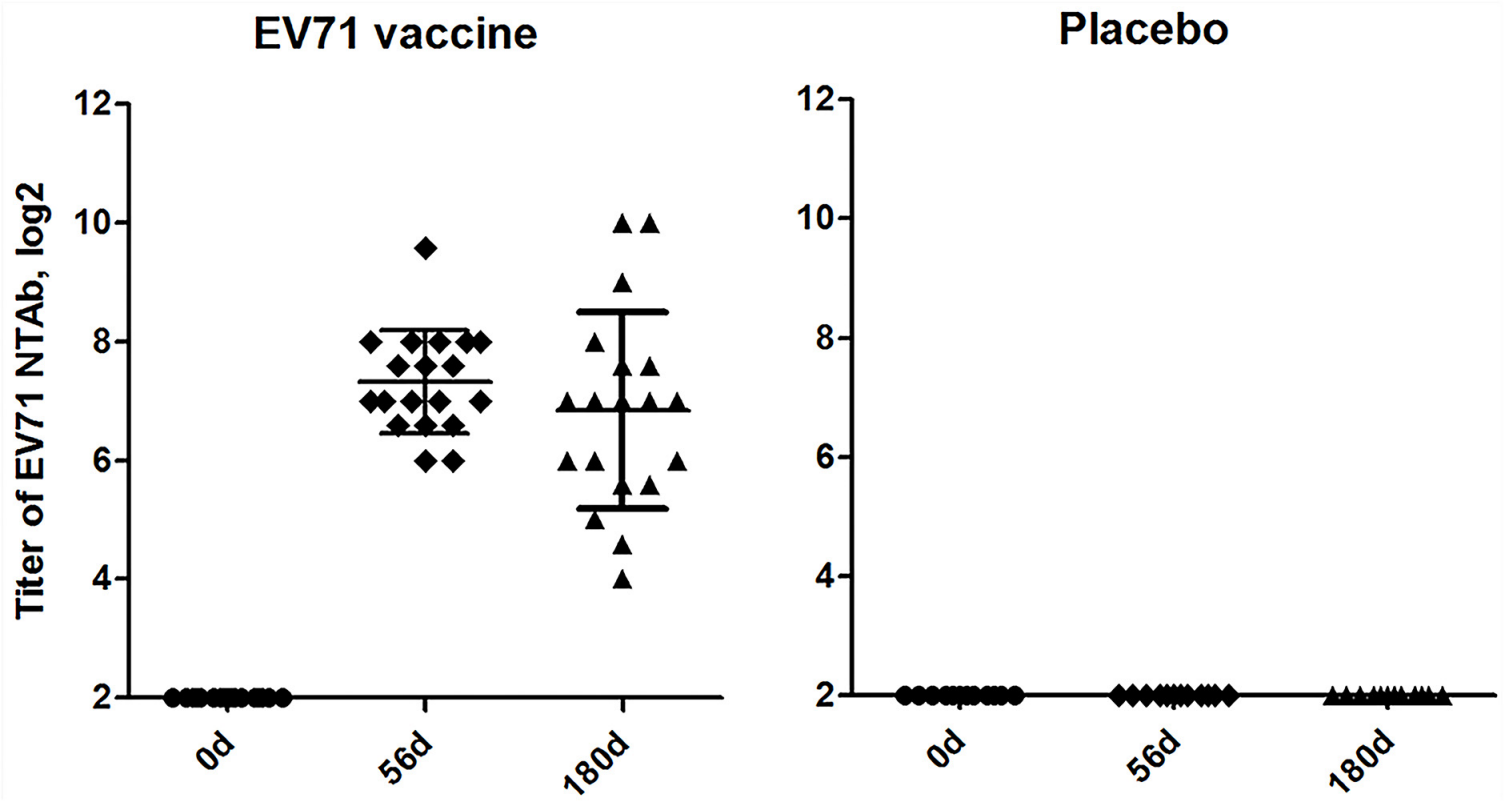
Titers of neutralizing antibodies responses to EV71 vaccine on days 0, 56 and 180 post vaccination. Each symbol represents an individual donor; median value and standard deviation were indicated. There were 19 subjects in the EV71 vaccine group and 13 in the placebo group. After vaccination (the first vaccination on day 0 and boosted on day 28), seroconversion was 100% in EV71 vaccine group.

As shown in Fig 1, NTAb titers were detected on day 56 (56d) and 180 (180d) after initial inoculated with inactivated EV71 vaccine. NTAb seroconversion rates reached 100% at 56d and persisted up to 180d. The geometric mean titer (GMT) of anti-EV71 NTAb elicited by EV71 vaccine was 1:160 at 56d and 1:86 at 180d. NTAb titers in placebo group were both negative before and after vaccination.

### 2. Comparison of the gene expression patterns between primary and recall immune response

#### 2.1 Gene expression patterns in primary response

To find changes of gene expression in primary response, we compared gene expression levels in pre-immunization PBMCs stimulated by EV71 Ag. After filtration, we observed that overall 220 of 35035 probe sets were significantly and differentially up-regulated after stimulation(fold change≥1.5, *p*<0.05),

Using the DAVID Bioinformatics Database (http://david.abcc.ncifcrf.gov/), we analyzed the Gene Ontology (GO) terms of the 220 genes, which revealed an enrichment of genes related to various immunological responses, such as genes mediating response to virus, including ISG15, EIF2AK2, MX1, IFI27, MX2, PLSCR1, IFI35, TRIM22, IRF9, IFIH1, CCL22; genes involved in transcription, including UBTF, ZNF205, ELL2, IRF9, ZNF432, POLR3G, TMF1, ZNF431, ZNF665, ZNF417; immune response, including OAS1, IFI35, TRIM22, IFITM2, GBP1, CCL22; interspecies interaction between organisms, including ISG15, EIF2AK2, IFIH1, SF3B2; cell-cell signaling, including ISG15, BST2, POMC, CCL22 (Table B in S1 File).

**Fig 2:**
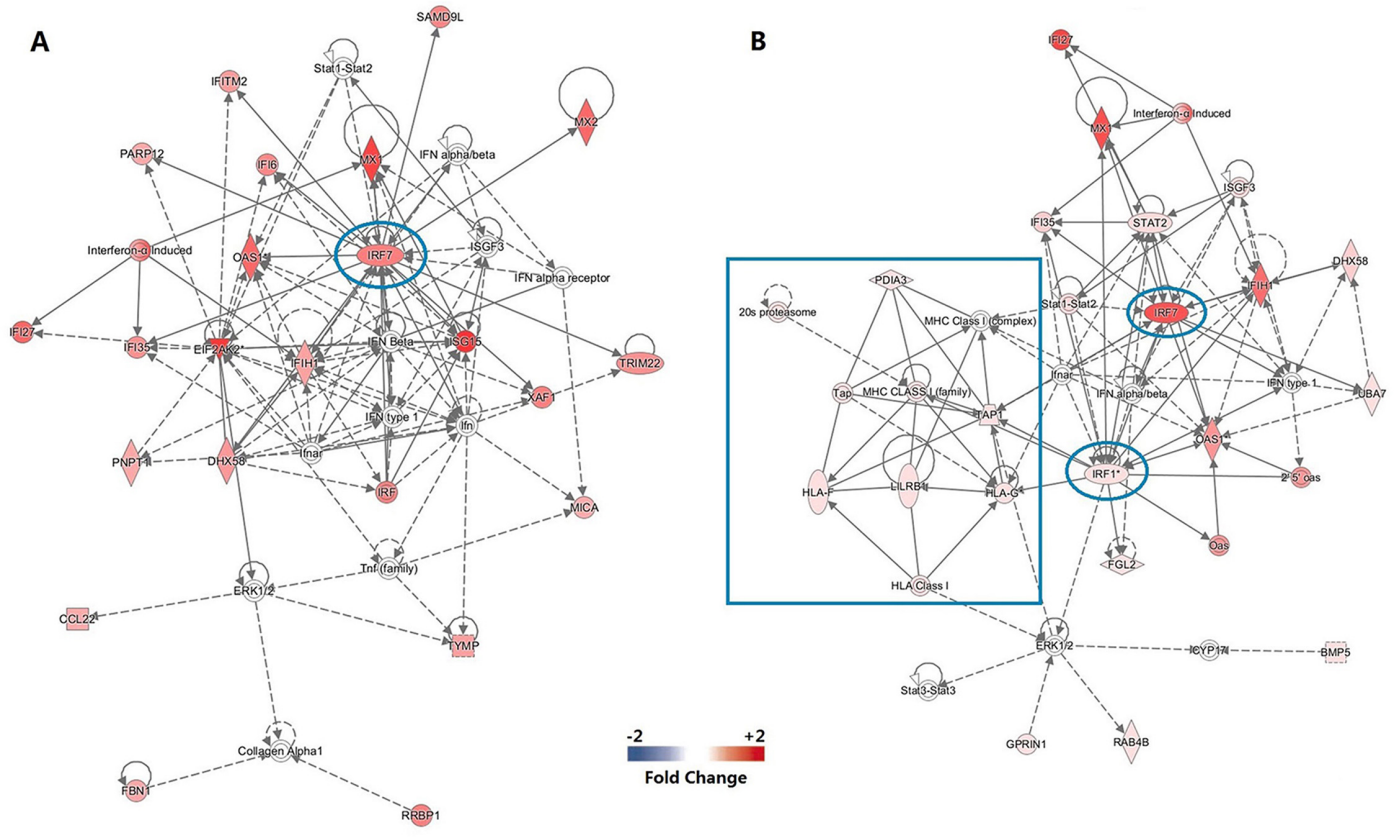
Molecular signatures of immune reponses to EV71 vaccine. (a) Ingenuity Pathways Analysis of DEGs in primary response. IRF7 was found as a hub of transcriptional regulation. (b) Gene network analysis of recall response. IRF7 was also found as a major node of transcriptional regulation. There was another key node, IRF1, which upregulated the genes related to activation of MHC-I as shown in the solid box.

Ingenuity Pathways Analysis revealed enrichment and activation of genes involved in Interferon Signaling (5 out of 34 genes were activated), Activation of IRF by Cytosolic Pattern Recognition Receptors (4/64), RIG1-like Receptors in Antiviral Innate Immunity (3/45) and Role of Pattern Recognition Receptors in Recognition of Bacteria and Viruses (4/127). Visualization of gene networks revealed a closely interacting network, including dozens of type I interferon (IFN-I) and antiviral genes. Among these, interferon regulatory factor 7 (IRF7), which is a major node of transcriptional network regulates downstream target antiviral genes, such as: ISG15, MX1, IFI27. Furthermore, the activation of IRF7 induced by EV71 Ag was observed to activate MDA-5 and LGP2, which are 2 members of RIG-I-like receptors (RLRs) (Fig 2A).

#### 2.2 Gene patterns in recall response

The recall immune responses were investigated by comparing post-vaccination gene expression patterns of PBMCs stimulated by EV71 Ag. After data filtration, we observed that 565 of 35035 probe sets met the fold change criteria (fold change≥1.5, *p*<0.05). Among these genes, 487 were up-regulated and 78 were down-regulated.

We analyzed the GO terms of the 565 genes, and results showed 89 of the 487 significantly up-regulated probe sets were identified within the top 5 GO terms including immune response, transcription inflammatory response,signal transduction,response to virus (Table C in S1 File). The down-regulated probe sets were identified within some metabolic pathways, such as lipid metabolism, cell wall catabolism and protein amino acid glycosylation.

Ingenuity Pathways Analysis revealed that differentially expressed genes (DEGs) were mainly involved in Interferon Signaling (9/34), Activation of IRF by Cytosolic Pattern Recognition Receptors (8/64), and Role of RIG1-like Receptors in Antiviral Innate Immunity (5/45). Gene networks analysis revealed that IRF7 also canactivate MDA-5 and LGP2, and played as a node of transcriptional regulation network in recall response. Several antivirus-genes were upregulated by IFR7 such as:MX1, IFI27 and IFIH1, similar with those in primary response. Another node of transcriptional regulation observed in recall immune response was IRF1. It directly regulated the expression of genes related to activation of MHC-I, including: PDIA3, TAP1, HLA-F, HLA-G, and LILRB1. These genes took a part in the activation of cytotoxic T cells (Fig 2B).

Expression level of MX1 mRNA had a high correlation with the NTAb level at 180d after vaccination (r = 0.81).

#### 2.3 Comparison between primary and recall immune response

In order to explore the similarities and differences between primary and recall immune response induced by EV71 Ag, a comparative analysis was carried out between them for the DEGs. By comparing, 124 DEGs were observed both in primary response and recall response; 96 DEGs were observed only in primary response; and 441 DEGs were observed only in recall response.

**Fig 3:**
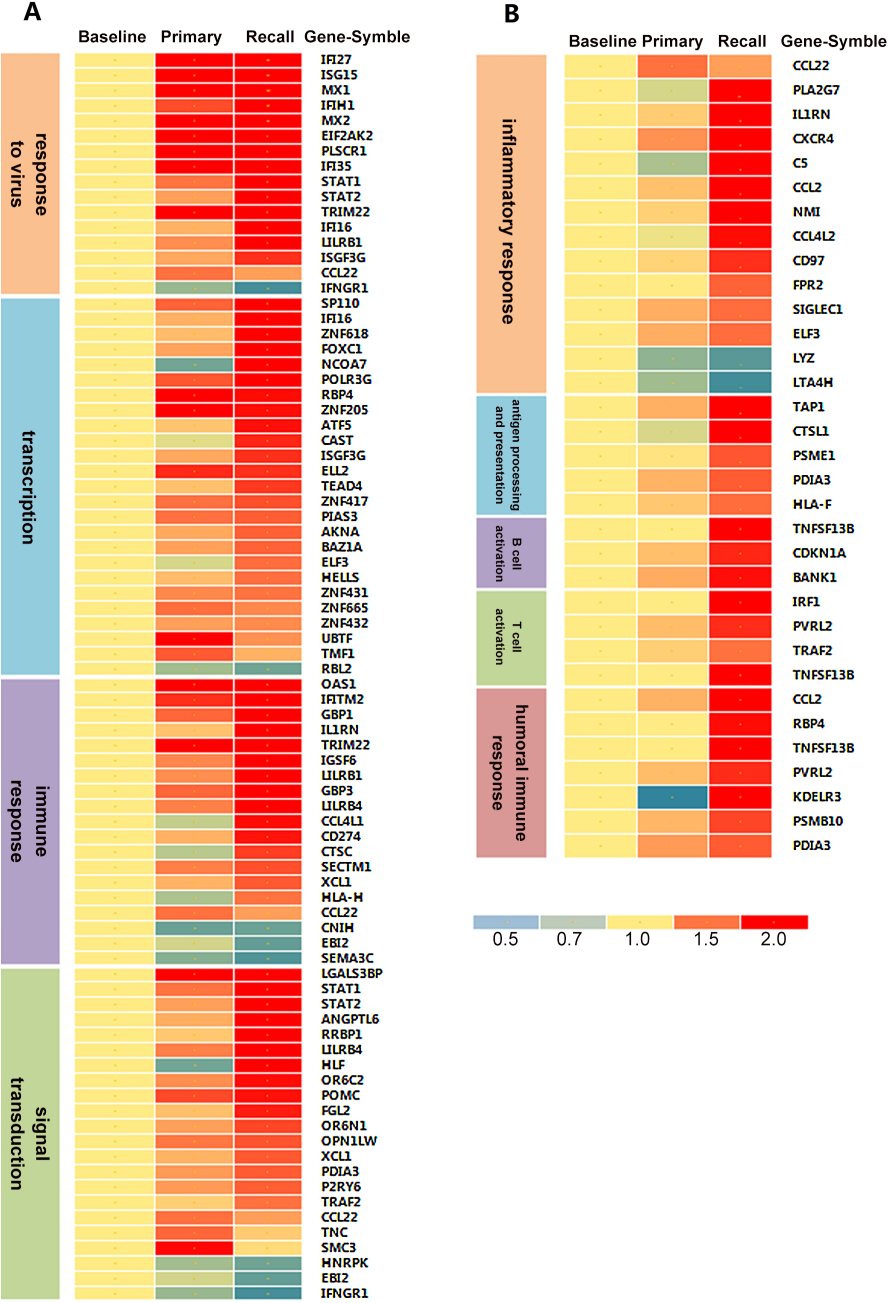
Heat map of DEGs in primary and recall response. Colors ranging from blue to red corresponded represent the DEGs’ average fold change among the subjects (n = 19). (a) Common genes identified in primary and recall response. However, the fold change of these genes in recall response was higher than that in primary response. (b) Pathways that were only observed in recall response,including inflammatory response, antigen processing and presentation, B cell activation, T cell activation and humoral immune response.

We analyzed the GO terms of the 124, 96 and 441 genes respectively. Results showed that the 124 genes both activated in primary and recall response were involved in response to virus, transcription, immune response and signal transduction. However, the fold change of these genes in recall response was higher than that in primary response (Fig 3A). Ninety-six genes observed only in primary response were involved in several metabolic pathways, such as lipid metabolism, fatty acid metabolism and DNA replication, but not immune response. 441 DEGs observed only in recall response involved in inflammatory response and the induction of an adaptive immune response, based on published literatures[19–33], such as TNFSF13B, CDKN1A and BANK1 involved in B Cell activation; IRF1, PVRL2, TNFSF13B and TRAF2 involved in T Cell activation;CCL2,RBP4,TNFSF13B,PVRL2,KDELR3,PSMB10 and PDIA3 involved in humoral immune response (Fig 3B).

Ingenuity Pathways Analysis revealed that the 124 common genes were enriched in Interferon Signaling, Activation of IRF by Cytosolic Pattern Recognition Receptors and Role of RIG1-like Receptors in Antiviral Innate Immunity. The visualization of gene networks showed a similar interacting network containing dozens of IFN-I regulated and antiviral genes both in primary response and recall response, and IRF7 played as a node of transcriptional regulation of these genes (Fig 2A & 2B). One of the most significant distinctions between networks of primary and recall immune response was that genes involved in MHC-I activation regulated by IRF1 were only observed in the recall response. This may be associated with the effect of the cytotoxic T cells (Fig 2B).

### 3. Confirmation of a subset of DEGs by quantitative RT-PCR

**Fig 4:**
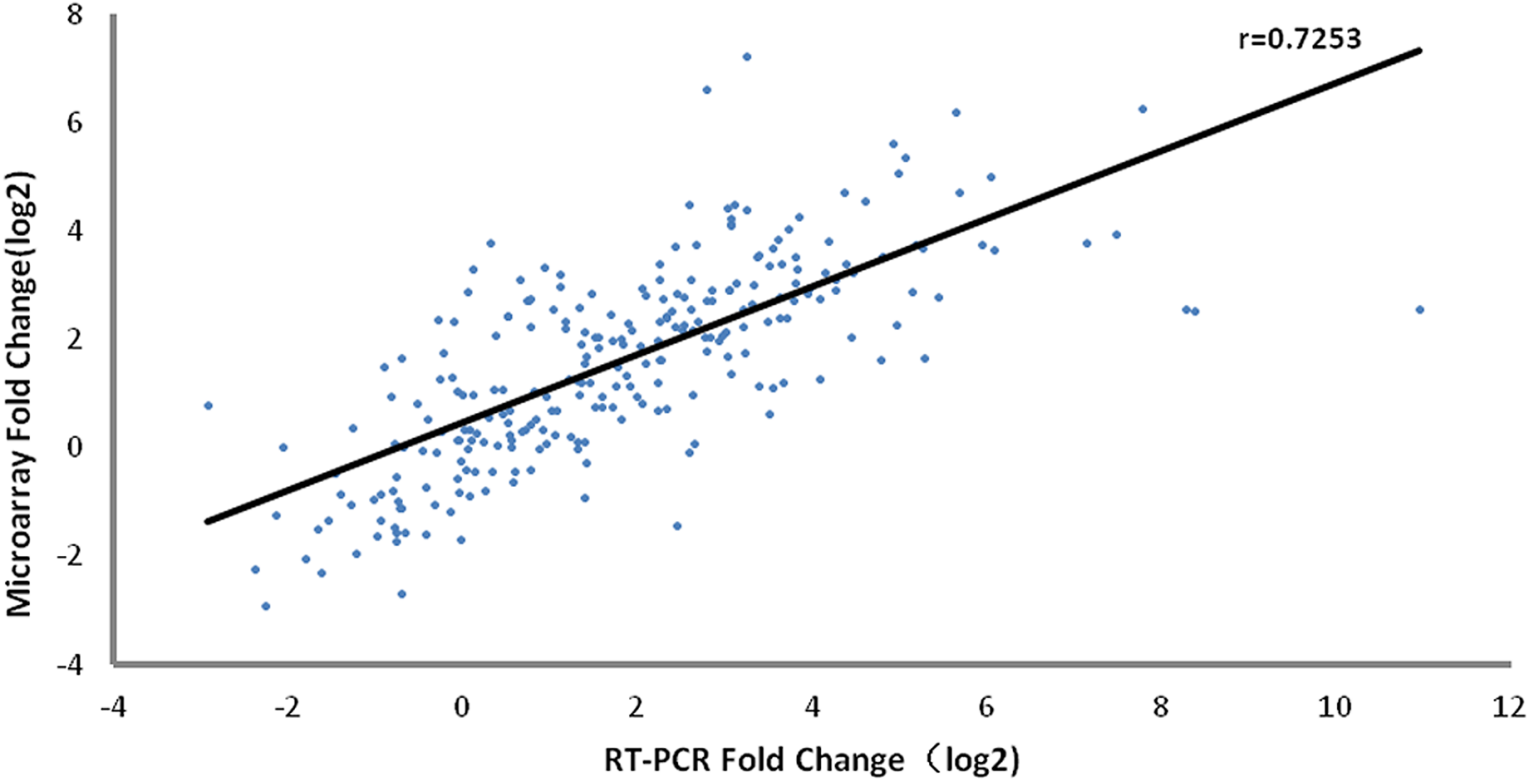
Verification of selective genes with RT-PCR. Correlation analysis of gene expressions changes with microarray and RT-PCR (r = 0.7253). 17 genes were selected to confirm within 60 samples. Each point represents a single gene at a given sample.

To evaluate the expression profiles of the genes detected in the microarray assay, RT-PCR verification was performed for 17 genes (defined in Materials and Methods) using samples from microarray analysis of vaccine recipients (n = 60). As shown in Fig 4 and Table D in S1 File, the expression pattern of the selected genes was consistent with the microarray data. A significant correlation (r = 0.7253) existed between the microarray data and RT-PCR results, suggesting that the microarray assay was reliable.

### 4. Cytokine secretion profiles in primary and recall immune response

In order to compare the cytokine secretion profiles between primary and recall response, cytokines in culture supernatants of PBMC after stimulation with EV71 Ag were tested by CapitalBio protein array which had a capacity of measuring 274 cytokines.

**Fig 5:**
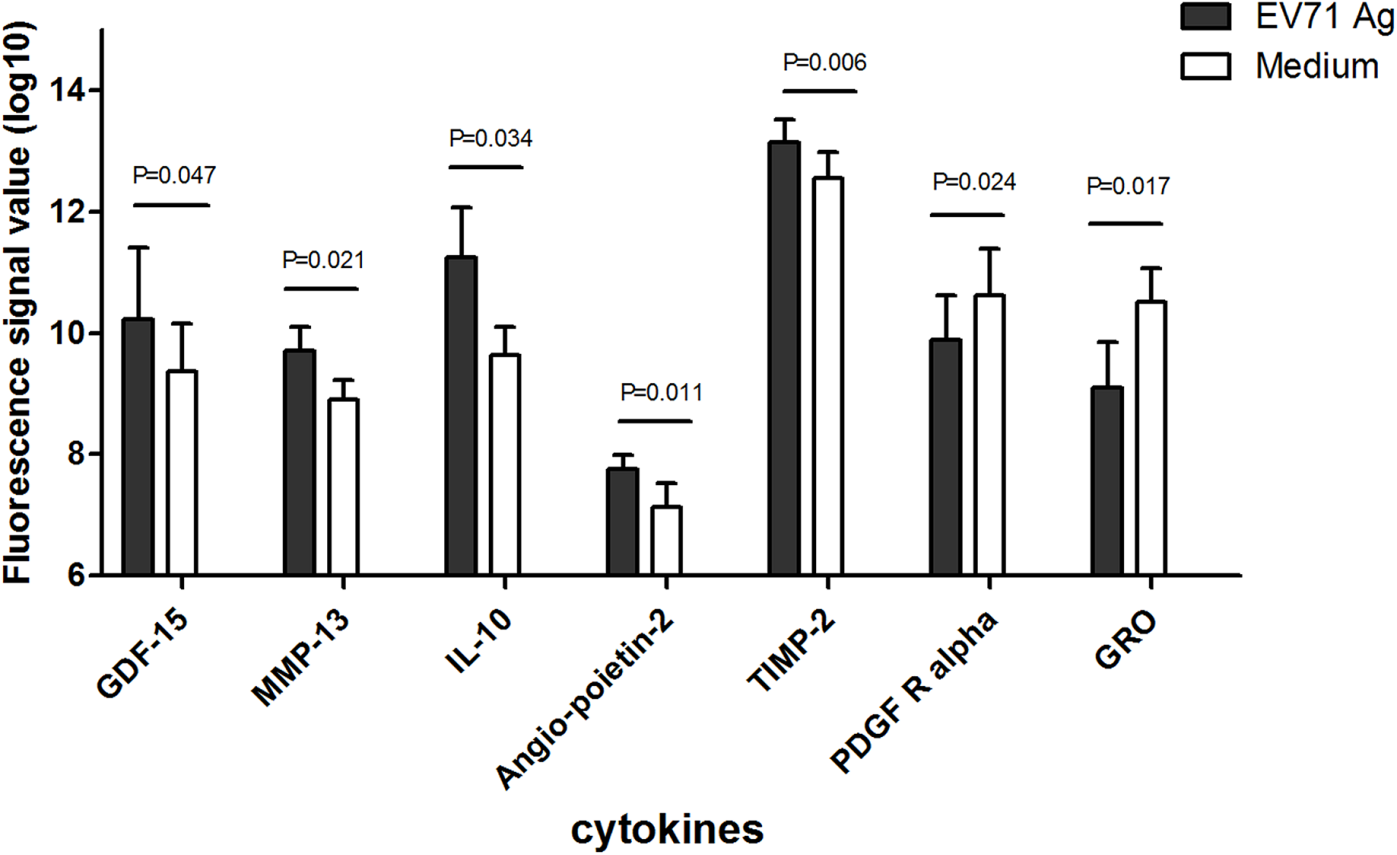
Cytokines profiling of *in vitro* EV71 antigen stimulated PBMCs which were from recipients before vaccination. PBMC were incubated with either EV71 antigen or medium as indicated in Materials and Methods. Five were up-regulated cytokines (GDF-15, MMP-13, IL-10, TIMP-2 and Angiopoietin-2) and 2 were down-regulated cytokines (PDGF-R alpha and GRO). Seven cytokines were significantly induced by EV71 antigen compared with control (P <0.05).

**Fig 6:**
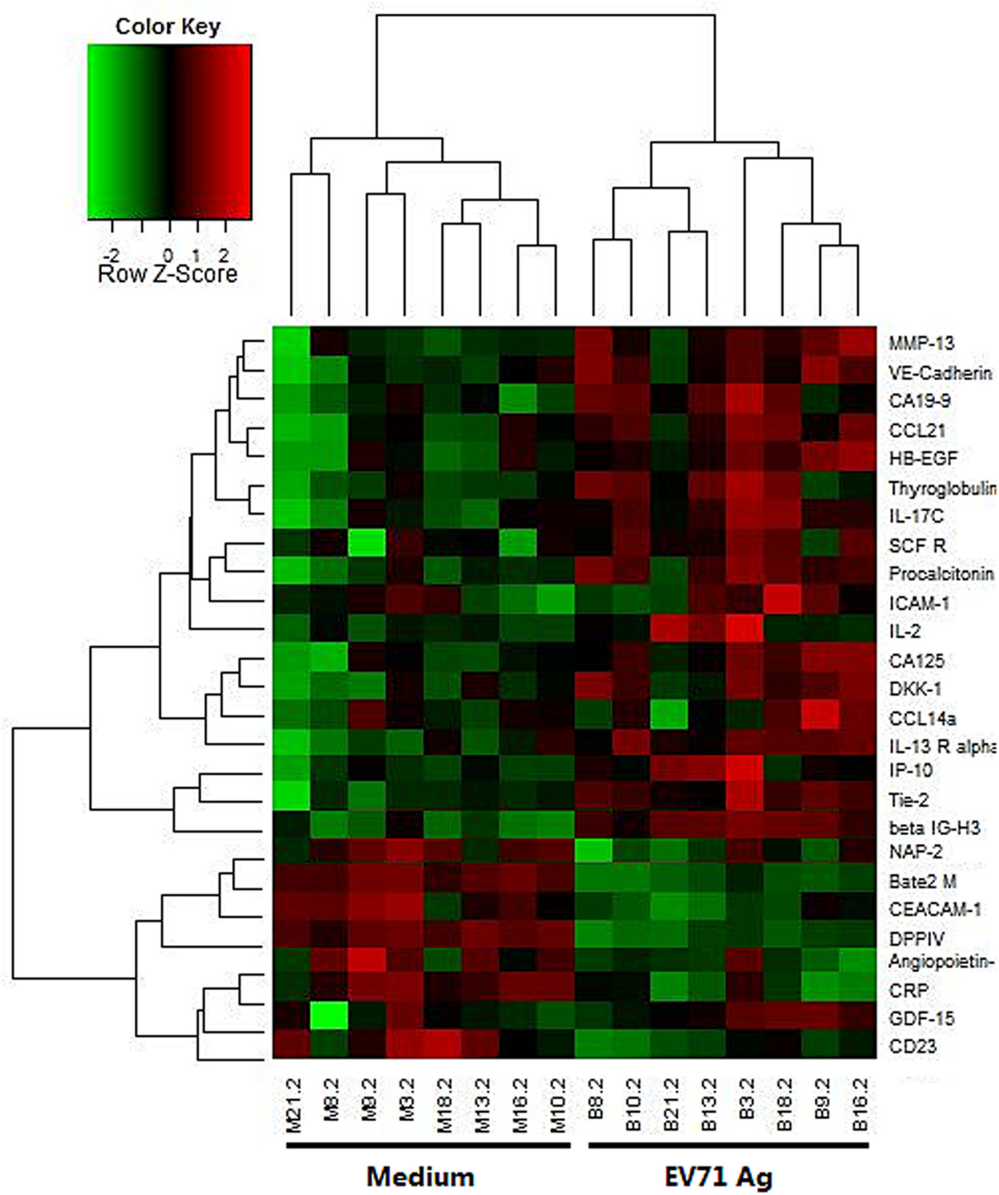
Cytokines profiling of *in vitro* EV71 antigen stimulated PBMCs which were from recipients after vaccination. PBMC were incubated with either EV71 antigen or medium as indicated in Materials and Methods. Up-regulated cytokines are shown in red and down-regulated cytokines in green. Each row represents a probeset and each column represents an individual.

Our results showed that 7 of 274 cytokines met the fold change criteria in primary response. Of these, 5 were up-regulated, including GDF-15, MMP-13, IL-10, TIMP-2 and Angiopoietin-2; and 2 were down-regulated, including PDGF-R alpha and GRO (Fig 5). Twenty-seven cytokines were found to be significantly differentially secreted in recall response. Among these 20 were up-regulated, such as Th1 type cytokines IL-2 and IP-10; competitive inhibitor of Th1 type cytokine receptor IL-13Ralpha2; chemokines CCL14a and CCL21 and so on. Seven were down-regulated,such as CD23, CRP, DPPIV, Bate2M and NAP-2 (Fig 6).

A correlation analysis between the level of cytokine secretion and the NTAb at 180d after vaccination showed that IP-10 had a high correlation coefficient with the antibody titers (r = 0.99).

## Discussion

“EV71 vaccine is an invaluable gift for children in the Asia-Pacific regions and worldwide”[34]. Exploring the effective protective immune mechanism will contribute greatly to the development of novel vaccines and the improvement of existing vaccines. To have a comprehensive understanding of the EV71 vaccines-induced immune response, the recall immune response has been explored as well as the primary immune response in this study.

Our results show that IFN-I and antiviral immune response were activated in both primary and recall response. The activation of IFN-I pathway may be necessary to the protective immune response elicited by EV71 vaccine. Because we have already found IFN-I related genes were generally activated on the early stage after vaccination with 5 different kinds of vaccines by a meta-analysis[35], and IFN-I contributes significantly to the procedure that mature B cells differentiate into antibody-secreting plasma cells[36]. IRFs are master regulators for IFN-I and antiviral genes signalling by Toll-like receptors and other cytosolic pattern-recognition receptors (PRRs)[37]. In our study IRF7 were identified as a node prominently involved in regulating transcription of interferon and antiviral genes in both primary and recall response. IRF7 governs both the systemic production of IFN in innate immune response and the local action of IFN from plasmacytoid dendritic cells in adaptive immunity[38]. Similar regulatory role of IRF7 was also observed in the immune response to yellow fever vaccines[10–11] and seasonal influenza vaccine[12].

More genes are activated in recall response to EV71 vaccine compared with the primary response. A dozen of them are enriched in activation of MHC class I, which is probably associated with the effect of memory CD8^+^ T cells. IRF1, another member of IRFs, is indentified as a master regulator of these genes. IRF1 could promote CD8^+^T cell maturity and induce a shift of the Th subset balance to a Th1 dominant state during Listeria infection, as well as activating expression of the cytokine Interferon beta like IRF7[39]. The activation of genes involved in inflammatory response was indentified in our study, which might assist in the effect of memory CD8^+^ T cells[40]. Other specific up-regulated genes involved in generating Ab procedure, such as B-Cell activation, antigen processing and presentation and humoral immune response, are important in generating high titer of antibodies after the boost.

The profiles of cytokines secretion were consistent with our finding in genes. IL-10, a specific secreted cytokine in primary response, coincides exactly with the process that naive B cell differentiate into the plasma cells in primary response, because it serves as a down-regulator of inflammatory cytokines and an enhancer of B cell proliferation[41]. In addition, IL-10 is involved in the regulation of the JAK-STAT signaling pathway[42] which was observed activate in the DEGs pathway analysis. An increased secretion of IL-2, IP-10, CCL14a, and CCL21 is observed in recall response. IL-2 and IP-10 promote the differentiation of certain immature T cells into regulatory T cells, effector T cells and memory T cells[43–44], and CCL14a and CCL21 promotes the migration and adhesion of T lymphocytes[45–46]. The increased secretion of IL-2, IP-10, CCL14a, and CCL21 is consistent with the up-regulation of IRF1, inflammatory genes and genes involved in activation of CD8+ T cells observed in microarray analysis.

The mRNA expression level of MX1 and cytokine secretion level of IP-10 in recall response were strongly correlated with NTAb level at 180d after vaccination. MX1 is responsible for a specific antiviral state against dozens of virus infection[47]. IP-10 is regarded as a marker to evaluate the antigen-specific cytotoxic T-lymphocytes induced by EV71 vaccine, and it was significantly induced by TIV and EV71 infection[12,48]. But further studies are needed to verify if MX1 and IP-10 can be used as markers to predict the immune persistence of EV71 vaccines.

Our study compared the different molecular mechanisms between primary and recall immune response on a new EV71 vaccine. IFN-I and antiviral immune response were actived in both primary and recall response. However, the up-regulation of genes involved in memory CD8^+^ T activation, inflammatory response, B-cell activation and humoral immune response were only found in recall response. Furthermore, MX1 and IP-10 are identified as two potential markers to predict the immune persistence of EV71 vaccines.

There are two limitations in this study. The recall immune response on EV71 vaccine was explored by re-stimulating PBMCs in vitro in this study, which did not completely simulate to the response in vivo. In addition, bio-markers indentified in our study still need be verified in broader vaccination receivers.

## Supporting Information

S1 File RT-PCR primers (Table A).Enrichment of GO terms in primary response (Top 10) (Table B). Enrichment of GO terms in recall response (Top 10) (Table C). Confirmation of microarray results with real-time PCR (n = 60) (Table D). (DOC)

## Abbreviations

HFMD: hand foot and mouth disease
EV71: enterovirus 71
NTAb: neutralizing antibodies
CPE: cytopathic effect
IRF7: interferon regulatory factor 7
GO: Gene Ontology
DEGs: differentially expressed genes
PBMC: peripheral blood mononuclear cell
PRRs: pattern-recognition receptors
IFN-I: type I interferon
