# Supplementary material for: Comparing the Primary and Recall Immune Response Induced by a New EV71 Vaccine Using Systems Biology Approaches
Source: PLoS One. 2015 Oct 14;10(10):e0140515. doi: 10.1371/journal.pone.0140515 (PMC4605509; doi:10.1371/journal.pone.0140515)
Supplement: S1 File — (DOC) [file pone.0140515.s001.doc]

**Table A.** RT-PCR primers

| Gene Symbol |  | Primer（5’to 3’） |
| --- | --- | --- |
| GAPDH | Forward Primer | TGTTGCCATCAATGACCCCTT |
|  | Reverse Primer | CTCCACGACGTACTCAGCG |
| MX1 | Forward Primer | ATATTTCAGGGATCTGCTGGAGG |
|  | Reverse Primer | CTTCCGGTATGTCGACACCAT |
| IFIH1 | Forward Primer | GCATATGCGCTTTCCCAGTG |
|  | Reverse Primer | TGTTCATTCTGTGTCATGGGTTTG |
| MX2 | Forward Primer | TTTTAACCCTCTGGGGACGC |
|  | Reverse Primer | GACGTTTGCTGGTTTCCAAGA |
| IRF7 | Forward Primer | GCTCCTGAGAGGTAAGAGCC |
|  | Reverse Primer | CAAGGAGCCACTCTCCGAAC |
| EIF2AK2 | Forward Primer | AAAAATCAGGAGACCCTGGCTA |
|  | Reverse Primer | TCTTCCCGTATCCTGGTTGGA |
| ISG20 | Forward Primer | AAGAGCATCCAGAACAGCCT |
|  | Reverse Primer | AGCAAGGTAGTTGCTGTCCC |
| IFITM2 | Forward Primer | CATCCCGGTAACCCGATCAC |
|  | Reverse Primer | CCTGTCCCTAGACTTCACGG |
| NUP62 | Forward Primer | GGATCACCTTTGACTGAGCGA |
|  | Reverse Primer | TGCCACAACCCCAAACTACA |
| IL1RN | Forward Primer | CCTCCAAGCTCCATCTCCACT |
|  | Reverse Primer | GGCACAGCCATCTTTCATACA |
| STAT2 | Forward Primer | CGGGACATTCAGCCCTTTTC |
|  | Reverse Primer | CTCATGTTGCTGGCTCTCCA |
| IFI6 | Forward Primer | GCGGGTAAGGATGCAGGTAAG |
|  | Reverse Primer | CCATTCAGGATCGCAGACCAG |
| GBP2 | Forward Primer | AGAAAAGGAGTTAAGGCAGACGA |
|  | Reverse Primer | TATAGTTTTCCCTTTCCCTCTCCAT |
| IRF1 | Forward Primer | CACACAGGCCGATACAAAGC |
|  | Reverse Primer | TATCAGGGCTGGAATCCCCA |
| RGS1 | Forward Primer | TTGACTTCCGCACTCGAGAA |
|  | Reverse Primer | GTTCACCCAGGGAGCCATAC |
| FOS | Forward Primer | GGGGCAAGGTGGAACAGTTA |
|  | Reverse Primer | GTCTGTCTCCGCTTGGAGTG |
| CD9 | Forward Primer | GGGAAACGCTGAAAGCCATC |
|  | Reverse Primer | GCCAAATATCATGACCACGGC |
| IFNGR1 | Forward Primer | CCAAGTCCTTGATCTCTGTGGT |
|  | Reverse Primer | GGAGACAACGGCTCTTCACA |
|  |  |  |

**Table B.** Enrichment of Go terms in primary response（Top 10）

| GO Term | p-Value | Gene symbol |
| --- | --- | --- |
| Response to virus | 4.15E-27 | ISG15;EIF2AK2;MX1;IFI27;MX2;PLSCR1;IFI35;TRIM22;IRF9;IFIH1;CCL22 |
| Transcription | 1.39E-10 | UBTF;ZNF205;ELL2;IRF9;ZNF432;POLR3G;TMF1;ZNF431;ZNF665;ZNF417 |
| Regulation of transcription, DNA-dependent | 1.38E-09 | ZNF205;TRIM22;ELL2;IRF9;ZNF432;TMF1;ZNF431;ZNF665;ZNF417 |
| Immune response | 3.09E-08 | OAS1;IFI35;TRIM22;IFITM2;GBP1;CCL22 |
| Interspecies interaction between organisms | 5.46E-07 | ISG15;EIF2AK2;IFIH1;SF3B2 |
| Cell-cell signaling | 8.21E-06 | ISG15;BST2;POMC;CCL22 |
| DNA replication | 1.95E-05 | TYMP;RMI1;GINS4 |
| Signal transduction | 9.80E-05 | MX1;LGALS3BP;SMC3;POMC;TNC;CCL22 |
| DNA mediated transformation | 2.03E-04 | SMC3 |
| Response to type I interferon | 2.03E-04 | SP100 |

**Table C.** Enrichment of Go terms in recall response（Top 10）

| GO Term | p-Value | Gene symbol |
| --- | --- | --- |
| Response to virus | 1.65E-32 | IFI27;ISG15;MX1;IFIH1;MX2;EIF2AK2;ISG20;PLSCR1;IFI35;STAT1;STAT2;TRIM22;LILRB1;IRF9;CCL4;IFNGR1 |
| Immune response | 1.23E-28 | OAS1;IFITM2;GBP1;GBP5;IFI35;TRIM22;RGS1;GBP4;TAP1;GBP2;IGSF6;LILRB1;GBP3;LILRB4;CCL4;CD274;ERAP2;SECTM1;IKBKE;XCL1;HLA-G;GPR183;SEMA3C |
| Regulation of transcription, DNA-dependent | 2.40E-20 | HES4;STAT1;TRIM22;IRF1;ZNFX1;NCOA7;MYBL2;DRAP1;IRF9;ZNF432;HLF;ZNF205;E2F3;ELL2;ZNF417;PIAS3;ETV6;AKNA;HELLS;RCOR2;ZNF431;YEATS2;RBL2;PHF20 |
| Oxidation reduction | 3.61E-17 | GMPR;SCO2;CBR1;LOX;RRM2;SQRDL;GPD2;CYP4A11;DUS4L;PCYOX1;ALDH3A2;ALDH2;PHYH;HSD17B4 |
| Transcription | 5.18E-16 | IRF1;FOXC1;NCOA7;POLR3G;DRAP1;IRF9;ZNF432;HLF;ZNF205;ATF5;E2F3;ELL2;ZNF417;CRCP;PIAS3;ETV6;AKNA;HELLS;ZNF431;RBL2;PHF20 |
| Signal transduction | 3.44E-13 | MX1;LGALS3BP;STAT1;STAT2;ANGPTL6;TN13B_HUMAN;LILRB4;LTA;CCL4;OR6C2;POMC;FGL2;PTGER2;OR6N1;LYN;OPN1LW;XCL1;PDIA3;TRAF2;GPR183;IFNGR1 |
| Inflammatory response | 2.60E-12 | PLA2G7;C5;NMI;CCL2;CCL4;SIGLEC1;LYZ;LTA4H;FOS |
| Interspecies interaction between organisms | 9.01E-11 | ISG15;IFIH1;EIF2AK2;STAT1;STAT2;LYN;SF3B2;CFLAR |
| Cell adhesion | 3.21E-10 | LGALS3BP;SLAMF7;SELL;POSTN;CCL2;HAPLN3;CCL4;SCARB2;SPON1;CD9 |
| Anti-apoptosis | 5.93E-09 | FOXC1;CCL2;ATF5;BAG1;HELLS;CFLAR |

**Table D.** Confirmation of microarray results with real-time PCR (n=60)

| Gene | Gene Name | Mean Fold Change | |
| --- | --- | --- | --- |
|  |  | Microarray | RT-PCR |
| IFNGR1 | Interferon-gamma receptor alpha chain precursor | 0.557034 | 0.663214685 |
| CD9 | Leukocyte antigen | 0.542999 | 0.730467987 |
| FOS | Proto-oncogene protein c-fos | 0.38058 | 0.518510371 |
| IRF1 | Interferon regulatory factor 1 | 2.920837 | 2.951224379 |
| ISG20 | Interferon-stimulated gene 20 kDa protein | 6.844702 | 11.27480704 |
| MX1 | Interferon-induced GTP-binding protein Mx1 | 19.8056 | 32.03904743 |
| MX2 | Interferon-induced GTP-binding protein Mx2 | 11.4343 | 13.6189711 |
| EIF2AK2 | Interferon-induced, double-stranded RNA-activated protein kinase | 8.586543 | 7.985507356 |
| RGS1 | Regulator of G-protein signaling 1 | 3.561132 | 6.247493218 |
| IRF7 | Interferon regulatory factor 7 | 12.42653 | 5.600420924 |
| STAT2 | Signal transducer and activator of transcription 2 | 3.999206 | 5.909509336 |
| IFITM2 | Interferon induced transmembrane protein 2 | 3.95894 | 3.754479041 |
| IFIH1 | Interferon-induced helicase C domain-containing protein 1 | 10.28015 | 7.049847637 |
| IFI6 | Interferon-induced protein 6-16 precursor | 3.593821 | 140.5646715 |
| NUP62 | Interleukin- 4-induced protein | 4.017416 | 1.406455386 |
| IL1RN | Interleukin-1 receptor antagonist protein precursor | 3.832182 | 25.38004881 |
| GBP2 | Guanylate-binging protein 2 | | 1.618681 | | --- | | | 4.766510999 | | --- | |
